# Characterization and Attenuation of Streptozotocin-Induced Diabetic Organ Damage by Polysaccharides from Spent Mushroom Substrate *(Pleurotus eryngii)*

**DOI:** 10.1155/2018/4285161

**Published:** 2018-09-30

**Authors:** Min Liu, Wangjinsong Yao, Fulan Zhao, Yongfa Zhu, Jianjun Zhang, Hui Liu, Lin Lin, Le Jia

**Affiliations:** ^1^College of Life Science, Shandong Agricultural University, Tai'an 271018, China; ^2^State Key Laboratory of Crop Biology, College of Life Science, Shandong Agricultural University, Tai'an 271018, China; ^3^The First Peoples' Hospital of Tai'an, Tai'an 271000, China

## Abstract

The aim of this work was to characterize spent mushroom substrate polysaccharides (MSP) from *Pleurotus eryngii* and their antioxidant and organ protective effects in streptozotocin- (STZ-) induced diabetic mice. The enzymatic-, acidic-, and alkalic- (En-, Ac-, and Al-) MSP were extracted from *P. eryngii* with snailase (4%), hydrochloric acid (1 mol/l), and sodium hydroxide (1 mol/l), respectively. The characterizations were evaluated by spectral analysis. In animal experiments, the enzymatic activities, lipid peroxide contents, and serum lipid parameters were measured, and histological observations of the liver, kidney, pancreas, and heart were conducted. The results demonstrated that treatment with En-, Ac-, and Al-MSP increased the organ enzymatic activities, decreased the organ lipid peroxide contents, mitigated the serum biochemistry values, and ameliorated the histopathology of diabetic mice, indicating that En-, Ac-, and Al-MSP could potentially be used as functional foods for the prevention of diabetes.

## 1. Introduction

Diabetes mellitus (DM), an endocrine metabolic disease characterized by a group of complex, chronic symptoms, is usually considered the major health risk worldwide due to its stimulation of health complications including heart disease, blindness, and organ failure [1, 2]. It has been reported that approximately 552 million people will suffer from DM by the year 2030 [3]. At present, several studies have reported that the occurrence and progression of DM and its complications involve many factors, including hemodynamic disorders, genetic predisposition, and disorders of biochemical metabolism [4]. However, the detailed mechanisms remain poorly understood. A mouse model of DM, induced by a single intravenous injection of streptozotocin (STZ), has been well documented to exhibit hyperglycemia similar to that in human patients [5]. Clinically, synthetic antidiabetic drugs have been widely used in DM therapy but cause side effects and toxicity during long-term use [6]. Hence, it is vitally important to seek natural and nontoxic antioxidants to prevent and treat DM. Therefore, the focus has recently shifted to identifying harmless natural antioxidants from edible materials [7].

Currently, the popularity of health foods has increased due to their abundant bioactivities [8, 9]. As species widely used in traditional Chinese medicines, mushrooms have drawn increasing attention for their multiple pharmacological effects, such as the immunomodulatory properties of heteroglycan from *Pleurotus ferulae* [10], the neuroprotective effects of *p*-terphenyls from *Polyozellus multiplex* [11], and the antioxidant, antidiabetes, antidementia, and anti-inflammatory effects of fruiting body extracts from *Trametes pubescens* [12]. As the most abundant substance in mushroom extracts, polysaccharides have potential activities in maintaining these pharmacological effects [13]. *Pleurotus eryngii*, known as the king oyster mushroom, has received much interest from consumers due to its attractive flavor and high nutritional value [14]. In addition, it has been documented that the polysaccharides extracted from *P. eryngii* have multiple bioactivities, including antioxidant, hypolipidemic, and hypoglycemic activities and hepatoprotective and tumor inhibitory effects [13, 15].

Spent mushroom substrate (SMS) is composed of fungal mycelia, extracellular hydrolytic enzymes, and partially modified lignocellulosic substrates [16]. The mushroom cultivation industry produces tons of SMS, which is usually discarded as a surplus waste product after fruiting body harvest, leading to low-efficiency utilization. Worse, the overaccumulation of SMS can cause serious environmental problems, such as eutrophication [17]. The environmentally sound disposal of this residue incurs significant costs for the industry, besides which the rate of SMS production simply exceeds its demand from existing applications [18]. However, previous studies have demonstrated that polysaccharides can be extracted from SMS. Hence, the exploration of SMS as well as the utilization of SMS-derived and value-added products seems to be of significant potential [2]. The influence of mushroom spent substance polysaccharides (MSP) on multiple organ failure in DM or on the factors that contribute to the attenuation of endothelium-dependent relaxation in DM animals has not yet been clearly elucidated. The aim of present work was to assess the antioxidation and attenuation of organ damage on the liver, kidney, pancreas, and heart by enzymatic-, acidic-, and alkalic-MSP (En-, Ac-, and Al-MSP) from SMS of *P. eryngii*, for analyzing the possible antidiabetes mechanisms with oxidative factors and providing health benefits in foods and antidiabetic drugs pharmaceutically.

## 2. Material and Methods

### 2.1. Material and Chemicals

The fresh *P. eryngii* SMS was provided by Shandong Ronfun Mushroom Co. Ltd. (Dongying, China). The STZ was purchased from Sigma Chemicals Co. Ltd. (St. Louis, USA). The diagnostic kits were purchased from Nanjing Jiancheng Bioengineering Ins. (Nanjing, China). The ELISA kits were purchased from Jiangsu Mei Biao Biological Technology Co. Ltd. (Yancheng, China). All other chemicals were purchased from Beijing Solarbio Science and Technology Co. Ltd. (Beijing, China).

### 2.2. Preparation of En-, Ac-, and Al-MSP

The cleaned *P. eryngii* SMS removed the epibiotic fruiting bodies and was dried (55°C) and powdered. The three MSP were extracted from the residue by hot water extraction with snailase solution (4%, 1 : 4, *w*/*v*) at 37°C for 6 h, HCl (0.5 mol/l, 1 : 10, *w*/*v*) at 75°C for 6 h, and NaOH (0.5 mol/l, 1: 10, *w*/*v*) at 80°C for 6 h. The supernatant was mixed with ethanol (95%, 1 : 3) at 4°C for 24 h. After centrifugation (3600 rpm, 15 min), the precipitates were deproteinized by Staub [19] and the carbohydrate contents were determined by the phenol-sulfuric acid colorimetric method, using glucose as standard [20]. Finally, the deproteinated precipitates were pooled and lyophilized to yield En-, Ac-, and Al-MSP.

### 2.3. Polysaccharide Characterizations

The molecular weights of three polysaccharides were determined by high-performance liquid chromatography (HPLC) operated with a HPLC system (Shimadzu LC-2010AT, Japan) equipped with an Atlantis C18 column (250 mm × 4.6 mm × 5 *μ*m) and a refractive index detector. The deionized water was used as mobile phase at a flow rate of 1 ml/min, the column temperature was maintained at 30°C, and the injection volume was 20 *μ*l. The molecular weights were determined by the calibration curves with a series of standard dextrans.

The monosaccharide compositions were determined by gas chromatography (GC) (GC-2010, Shimadzu, Japan) equipped with a capillary column of Rtx-1 (30 m × 0.25 mm × 0.25 *μ*m). Briefly, the samples were hydrolyzed with trifluoroacetic acid (TFA, 2 mol/l, 110°C) for 4 h. After acetylating with hydroxylamine hydrochloride and pyridine, the hydrolyzed supernate (1 *μ*l) was injected into the column and equipped with flame ionization detector. Sugar identification was confirmed by comparison with standard monosaccharides of mannose, rhamnose, glucose, galactose, arabinose, ribose, and xylose. The relative molar ratios were calculated by the area normalization method according to the chromatogram.

The purity of the three polysaccharides was recorded with a UV-visible spectrophotometer (Hitachi UV-3010, Japan). The polysaccharides were dissolved and diluted to an appropriate concentration for the ultraviolet (UV) analysis, that is, within the range from 200 to 400 nm.

Spectroscopy analysis of Fourier-transform infrared (FT-IR) was determined with a 6700 Nicolet Fourier-transform infrared spectrophotometer (Thermo Co., Madison, WI, USA) within the range from 4000 to 400 cm^−1^, using KBr disc method to prepare the specimen.

^13^C and ^1^H nuclear magnetic resonance (NMR) spectroscopy experiments were conducted using a 700 MHz Varian Mercury 2010 Magneto Oxford spectrometer at 60°C, and the sample was dissolved in dimethyl sulfoxide (DMSO).

The morphological features of En-, Ac-, and Al-MSP were analyzed by scanning electron microscope (SEM) (S-4800, FE-SEM, Hitachi High-Technologies, Japan). The dried powder of the polysaccharides was affixed to a glass slide and coated with gold powder to make them conductive. Images were taken at a magnification of 1000x with an accelerating voltage of 10 kV.

The ultrastructure of three polysaccharides was analyzed by BioScope Catalyst atomic force microscope (AFM) (Bruker NanoScope, Billerica, MA) under ambient conditions. The polysaccharides were dissolved in distilled water at a concentration of 10 *μ*g/ml and filtered through a 0.45 *μ*m filter (NYL, 13 mm syringe filter, Whatman Inc., USA). The diluted solution (5 *μ*l) was dropped onto a freshly cleaved mica substrate and dried at room temperature. All specimens were scanned at a rate of 1.0 Hz per line in tapping mode. All images were 256 × 256 pixels.

### 2.4. Ethical Considerations

The experiments were performed and approved by the Institutional Animal Care and Use Committee of Shandong Agricultural University and in accordance with the Animals (Scientific Procedures) Act. 1986 (amended 2013).

### 2.5. Animal Experiments

Kunming strain mice (weighted 20 ± 2 g) were purchased from Shandong Taibang Co. Ltd. (Tai'an, China) and maintained at the Laboratory of Shandong Agricultural University. Mice were housed individually in cages in a room under controlled conditions (22 ± 2°C, 12 h/12 h light/dark cycle) for 5 days for acclimatization.

After acclimatization, the DM model was induced by intraperitoneal injection with STZ (80 mg/kg, freshly prepared in citrate buffer solution, 0.1 mol/l, pH 4.5) once daily for three days (24 h intervals) [21]. After 12 h of fasting, all STZ-injected mice were assessed by measuring the glucose (GLU) levels in the tail vein. Mice with GLU levels over 13.3 mmol/l were considered successful diabetic models [22]. Fifty successful diabetic mice were randomly divided into ten groups of five mice each, including one model control (MC) group and nine polysaccharide groups, using uninduced mice in the normal control (NC) group (*n* = 5). During the experimental procedure, NC and MC mice received only citrate buffer solution (0.1 mol/l, pH 4.5), while the mice in the polysaccharide groups were treated with En-, Ac-, and Al-MSP at 200, 400, and 800 mg/kg, respectively. The entire experiment lasted for 15 days, during which time the body weight was monitored daily.

At the end of the experiment, all the mice were fasted overnight and sacrificed under light ether anesthesia. Immediately, serum was obtained from the blood samples by centrifugation (10,000 r/min, 10 min, 4°C). The serum alanine transaminase (ALT) activities, aspartate aminotransferase (AST) activities, urea nitrogen (BUN) levels, creatinine (CRE) levels, albumin (ALB) levels, total cholesterol (TC) levels, triglyceride (TG) levels, high-density lipoprotein cholesterol (HDL-C) levels, very low-density lipoprotein cholesterol (VLDL-C) levels, and low-density lipoprotein cholesterol (LDL-C) levels were measured using an automatic biochemical analyzer (ACE, USA).

Meanwhile, the liver, kidney, pancreas, and heart were rapidly removed and weighed and homogenized (1 : 9, *w*/*v*) in phosphate buffer solution (4°C, 0.2 mol/l, pH 7.4). The organ supernatants were collected by centrifugation (10,000 rpm, 10 min), and the superoxide dismutase (SOD) and glutathione peroxidase (GSH-Px) activities, total antioxidant capability (T-AOC), and malondialdehyde (MDA) and lipid peroxide (LPO) contents of the supernatants were determined by using commercial reagent kits according to their instructions.

The tissue index was calculated as (tissue weight/body weight) (g/100 g body weight). The atherogenic index (AI) was calculated as (TC-HDL-C)/HDL-C [23].

The fresh tissue samples were immersed in paraformaldehyde solution (4%) for 24 h and embedded in paraffin. The thin sections (4-5 *μ*m thickness) were prepared on a microtome and stained with hematoxylin-eosin. Each section was photographed under a microscope to evaluate the histopathological changes (×400 magnifications).

### 2.6. Acute Toxicity Study

For the acute toxicity study, fifteen Kunming mice were divided into three groups (*n* = 5). The mice were gavaged daily for 15 days with En-, Ac-, and Al-MSP at a high dose of 4000 mg/kg [24, 25]. The gross behavioral changes, toxic symptoms, and mortality of these mice were observed over the course of the experiment.

### 2.7. Statistical Analysis

The results were expressed as the mean ± standard deviation (SD) to confirm the normal distribution. The data were analyzed by one-way analysis of variance (ANOVA) using SPSS statistical software package. Differences were considered significant at *P* < 0.05.

## 3. Results

### 3.1. Polysaccharide Characterizations

#### 3.1.1. Molecular Weights

The HPLC chromatograms indicated that the weight average molecular weight (Mw), number average molecular weight (Mn), and Z-average molecular weight (Mz) of En-MSP were 1.35 × 10^3^, 1.21 × 10^3^, and 1.33 × 10^3^ Da; the Mw, Mn, and Mz of Ac-MSP were 2.11 × 10^3^, 2.54 × 10^3^, and 2.19 × 10^3^ Da; and the Mw, Mn, and Mz of Al-MSP were 2.26 × 10^3^, 2.85 × 10^3^, and 1.93 × 10^3^ Da, respectively.

#### 3.1.2. Monosaccharide Compositions

**Figure 1:**
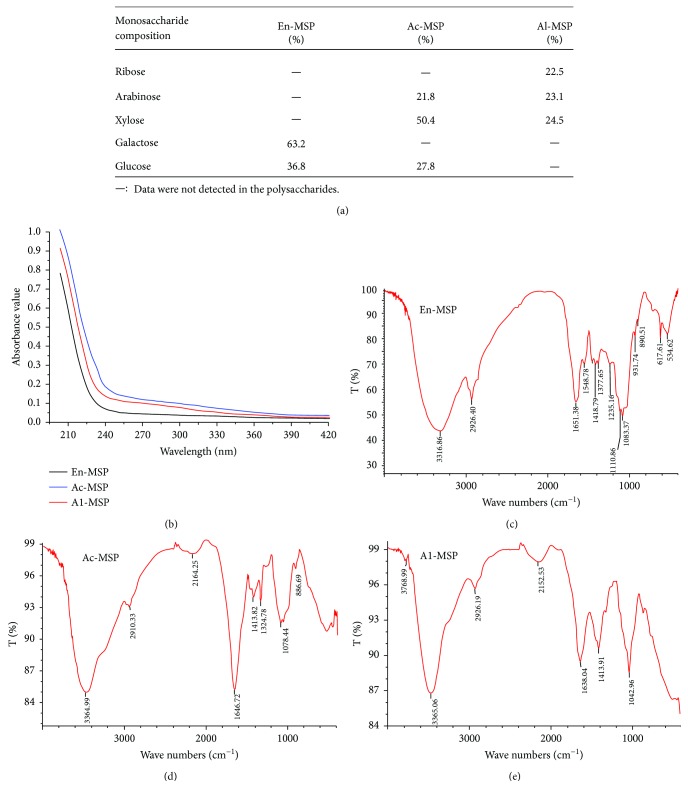
The monosaccharide compositions (a), UV spectrum (b), and FT-IR (c–e) analysis of En-, Ac-, and Al-MSP.

The monosaccharide composition of En-, Ac-, and Al-MSP was analyzed according to the retention time of standard monosaccharides (Figure 1(a)). It was clear that En-MSP consisted of two different monosaccharides, galactose and glucose, with a molar ratio of 3.51 : 2.04; Ac-MSP contained three monosaccharides, arabinose, xylose, and glucose, with a molar ratio of 1.50 : 3.40 : 1.54; and Al-MSP contained three monosaccharides, ribose, arabinose, and xylose, with a molar ratio of 1.50 : 3.53 : 1.63.

#### 3.1.3. UV and FT-IR Spectroscopy Analysis

UV spectroscopy of En-, Ac-, and Al-MSP showed that all three polysaccharides had no obvious absorption at 260 and 280 nm (Figure 1(b)), suggesting that no protein or nucleic acid was present in the samples.

FT-IR spectroscopy measurements were carried out to identify the structures of the polysaccharides including monosaccharide types, glucosidic bonds and functional groups (Figures 1(c)–1(e)). In the FT-IR spectra, there were nearly no differences in the functional group regions (4000–1400 cm^−1^) among the three polysaccharides. The broad peaks at about 3300 cm^−1^ of stretching vibration of the hydroxyl groups, and the peaks at about 2900 and 1600 cm^−1^ of carbon-hydrogen bonds, and the bands at about 1410 cm^−1^ of C-O (-COOH) stretching vibrations could be observed in En-, Ac-, and Al-MSP. Besides, the bands between 1000 and 1200 cm^−1^ indicated the three polysaccharides were all pyran rings. However, the difference of peaks numbers with En-MSP > Ac-MSP > Al-MSP were showed in the fingerprint regions. The absorptions at 890.51 and 886.69 suggested the presence of *α*-glucose in En-MSP and Ac-MSP. The band at 931.74 cm^−1^ indicated that *β*-galactose was only present in En-MSP [13, 26].

#### 3.1.4. ^1^H and ^13^C NMR Spectroscopy Analysis

**Figure 2:**
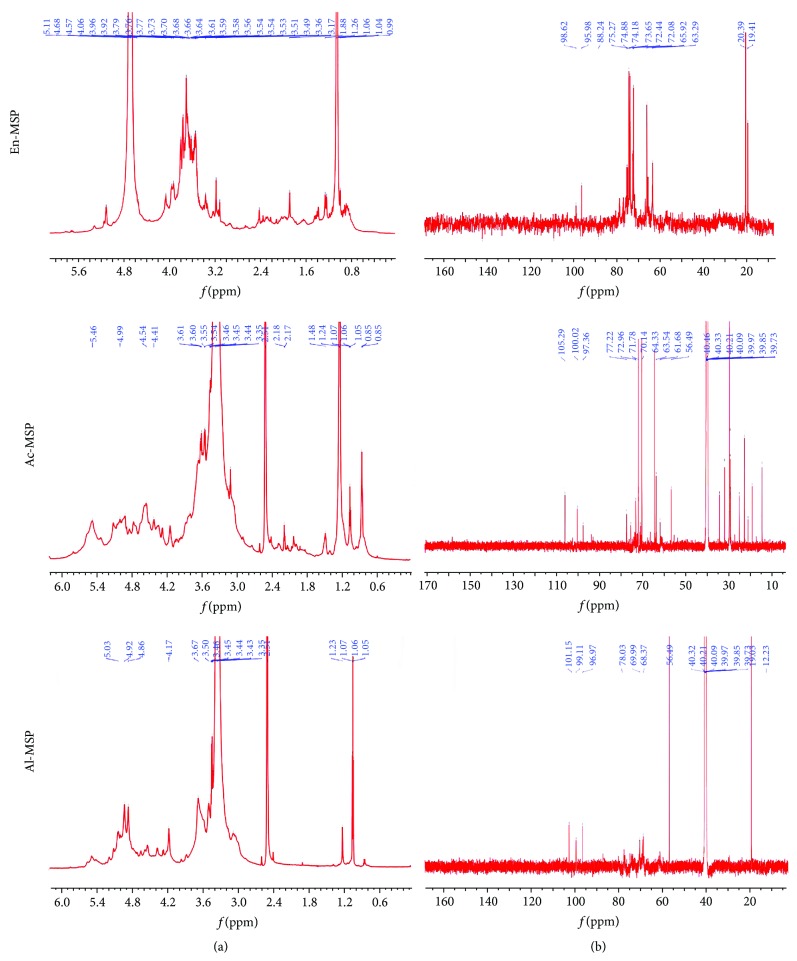
The ^1^H (a) and ^13^C (b) NMR analysis of En-, Ac-, and Al-MSP.

NMR is the most powerful instrument available for the identification of polysaccharide structures. ^1^H and ^13^C NMR spectroscopy was used to analyze the structures of En-, Ac-, and Al-MSP (Figures 2(a) and 2(b)). Complex groups of peaks in the spectra were observed for all three polysaccharides. In the anomeric region (4.3–5.9 ppm) of the ^1^H NMR spectrum, En-MSP showed two signals at 4.68 and 5.11 ppm, corresponding to anomeric protons (Figure 2(a)), suggesting the presence of two monosaccharide residues. Different patterns were observed in the anomeric region (4.3–5.9 ppm) of the other two polysaccharides. For Ac-MSP, three dominant signals appeared at 4.54, 4.99, and 5.46 ppm, suggesting the presence of three monosaccharide residues in Ac-MSP, while Al-MSP exhibited three monosaccharide residues at 4.86, 4.92, and 5.03 ppm. At the same time, in the anomeric carbon region (95–110 ppm) of ^13^C NMR spectrum, the En-MSP contained two signals at 95.98 and 98.62 ppm (Figure 2(b)), Ac-MSP had three signals at 97.36, 100.02, and 105.29 ppm, and Al-MSP had three signals at 96.97, 99.11, and 101.15 ppm, indicating that anomeric carbons were existed in the three polysaccharides [10].

#### 3.1.5. SEM and AFM Analysis

**Figure 3:**
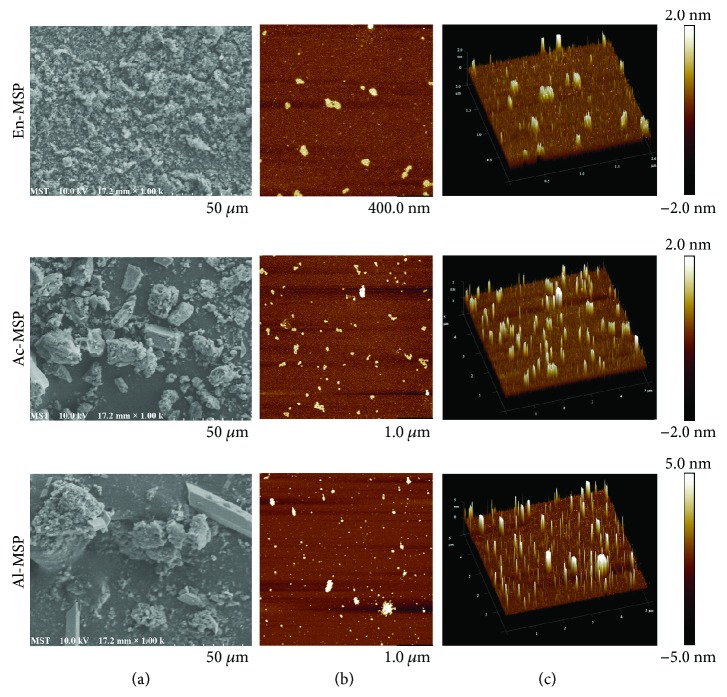
The SEM (a) and AFM (b-c) analysis of En-, Ac-, and Al-MSP.

SEM and AFM are powerful tools to analyze the conformation of macromolecules and the spatial structure and surface morphology of biomacromolecules on the nanometer scale to better understand their common physical properties [27]. Under 1000x magnification in SEM analysis, En-MSP showed an integrated surface with several regular shapes, and the particles were dispersed evenly (Figure 3(a)). The other two polysaccharides formed a rough surface with many small, lumpy particles and irregular pores. The images presented in Figures 3(b) and 3(c) are planar and three-dimensional representations from the AFM scans of the En-, Ac-, and Al-MSP. As seen in Figure 3, the conformations of the three polysaccharides are different. Clearly, the Ac-MSP adopted a structure primarily of random linear chains with a few spherical aggregates with diameters ranging from 200 to 500 nm and heights ranging from 0.5 to 2.0 nm, respectively, and Al-MSP formed aggregates with diameters ranging from 150 to 600 nm and heights ranging from 0.5 to 5.0 nm. In contrast, En-MSP appeared as spherical structures uniformly dispersed in aqueous solution, with dimensions ranging from 50 to 300 nm in length and from 0.5 to 2.0 nm in height.

### 3.2. Effects of MSP on GLU Levels and Body Weight

The effects of En-, Ac-, and Al-MSP on GLU levels and body weight in STZ-induced diabetic mice are displayed in Table 1. The initial GLU levels of all mice did not differ significantly. However, polysaccharide administration in STZ-induced diabetic mice led to a decrease in GLU levels after 15 d. Interestingly, oral administration of MSP for 15 days significantly decreased the GLU levels compared with those in the MC group (*P* < 0.05). En, Ac-, and Al-MSP administration at 800 mg/kg caused the most significant decreases in GLU levels, 44.92%, 42.25%, and 37.86%, respectively, compared with the levels in the MC group mice. Meanwhile, the body weights of mice were also investigated; these data are displayed in Table 1. Compared with the NC mice, the STZ-induced diabetic mice exhibited a significant decrease in body weight. Intriguingly, the administration of En-, Ac-, and Al-MSP at 800 mg/kg showed a superior effect on body weight loss, increasing the weight loss by 22.37%, 17.48%, and 13.02% in comparison with MC.

### 3.3. Effects of En-, Ac-, and Al-MSP on Serum Lipid Levels

**Figure 4:**
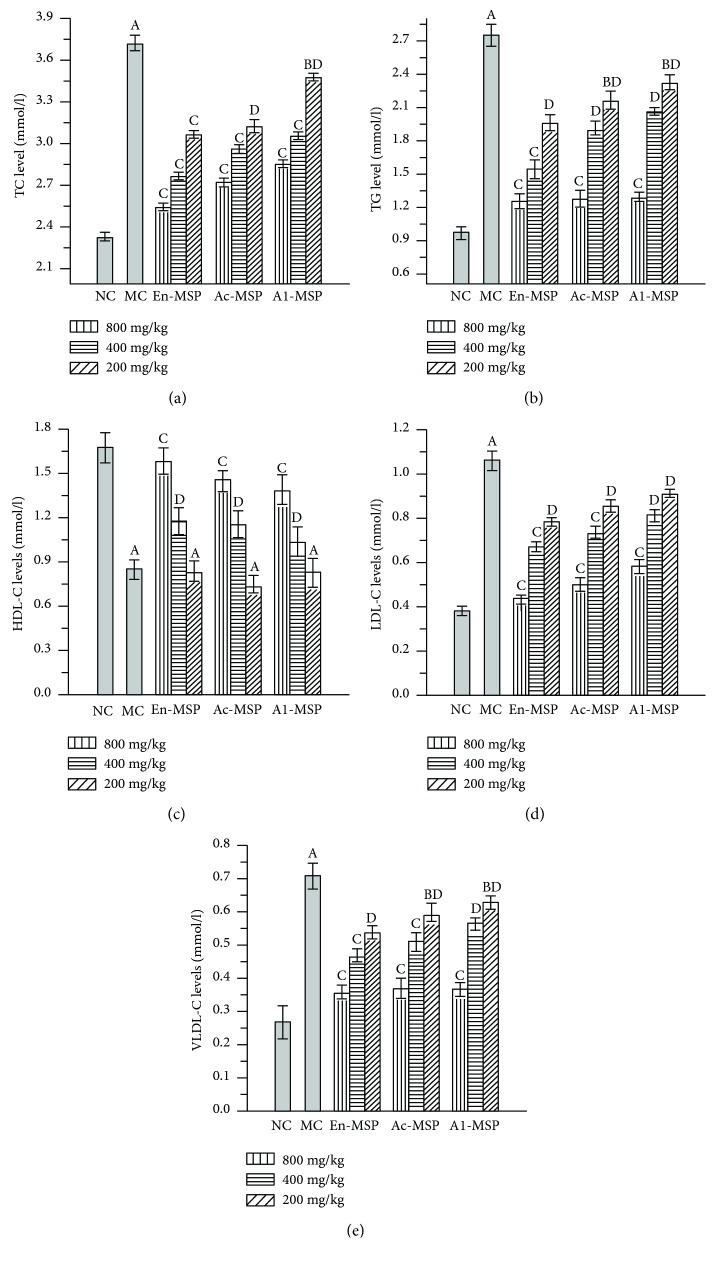
Effects of En-, Ac-, and Al-MSP on lipid properties. (a) TC levels, (b) TG levels, (c) HDL-C levels, (d) LDL-C levels, and (e) VLDL-C levels. The values are reported as the means ± SD (*n* = 5). (A): *P* < 0.01 and (B): *P* < 0.05 compared with the NC group, (C): *P* < 0.01 and (D): *P* < 0.05 compared with the MC group.

As shown in Figure 4, significant increases in TG, TC, LDL-C, and VLDL-C levels and significant decreases in HDL-C levels were observed in STZ-induced diabetic mice (the MC group) compared with the NC mice (*P* < 0.05), indicating that lipid dysfunction had occurred. Fortunately, treatment with polysaccharides (En-, Ac-, and Al-MSP) had a potent effect in suppressing the levels of TC, TG, LDL-C, and VLDL-C as well as increasing the levels of HDL-C. In particular, treatment with En-MSP at the dose of 800 mg/kg stimulated significant decreases in the TG, TC, LDL-C, and VLDL-C levels, of 54.54%, 31.72%, 59.43%, and 49.29%, respectively, and a significant increase in HDL-C levels, of 85.88%, relative to the levels in the MC group.

### 3.4. The Influence of En-, Ac-, and Al-MSP on Mouse Organs

**Figure 5:**
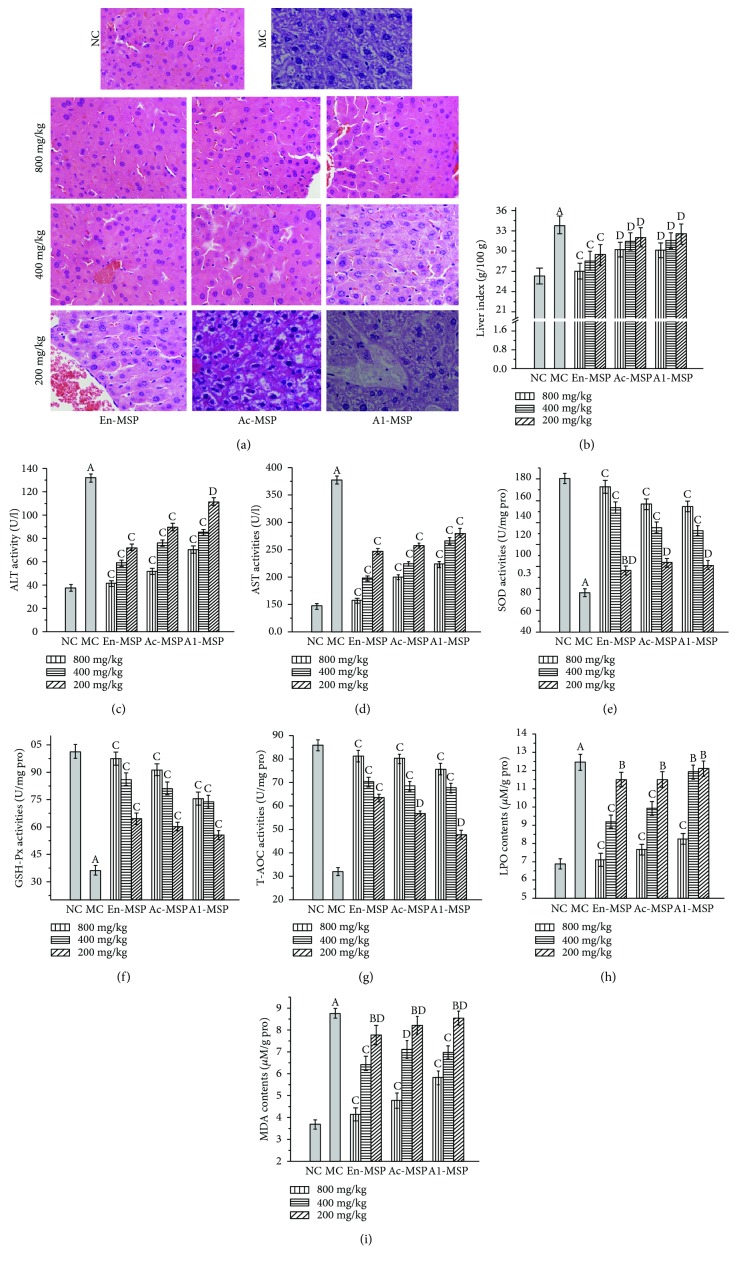
Effects of En-, Ac-, and Al-MSP on the (a) pathological observations, (b) liver index, (c-d) serum analysis, (e-g) hepatic enzymatic analysis, and (h-i) lipid peroxidation. The values are reported as the means ± SD (*n* = 5). (A): *P* < 0.01 and (B): *P* < 0.05 compared with the NC group, (C): *P* < 0.01 and (D): *P* < 0.05 compared with the MC group.

**Figure 6:**
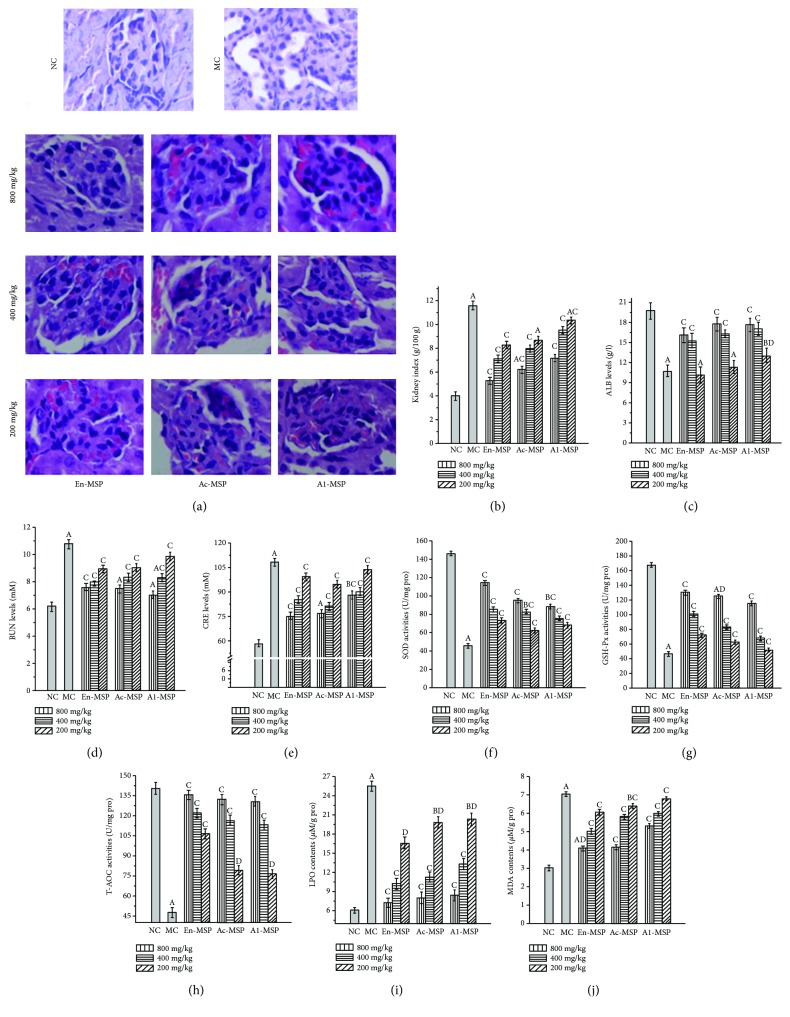
Effects of En-, Ac-, and Al-MSP on the (a) pathological observations, (b) kidney index, (c–e) serum analysis, (f–h) hepatic enzymatic analysis, and (i-j) lipid peroxidation. The values are reported as the means ± SD (*n* = 5). (A): *P* < 0.01 and (B): *P* < 0.05 compared with the NC group, (C): *P* < 0.01 and (D): *P* < 0.05 compared with the MC group.

**Figure 7:**
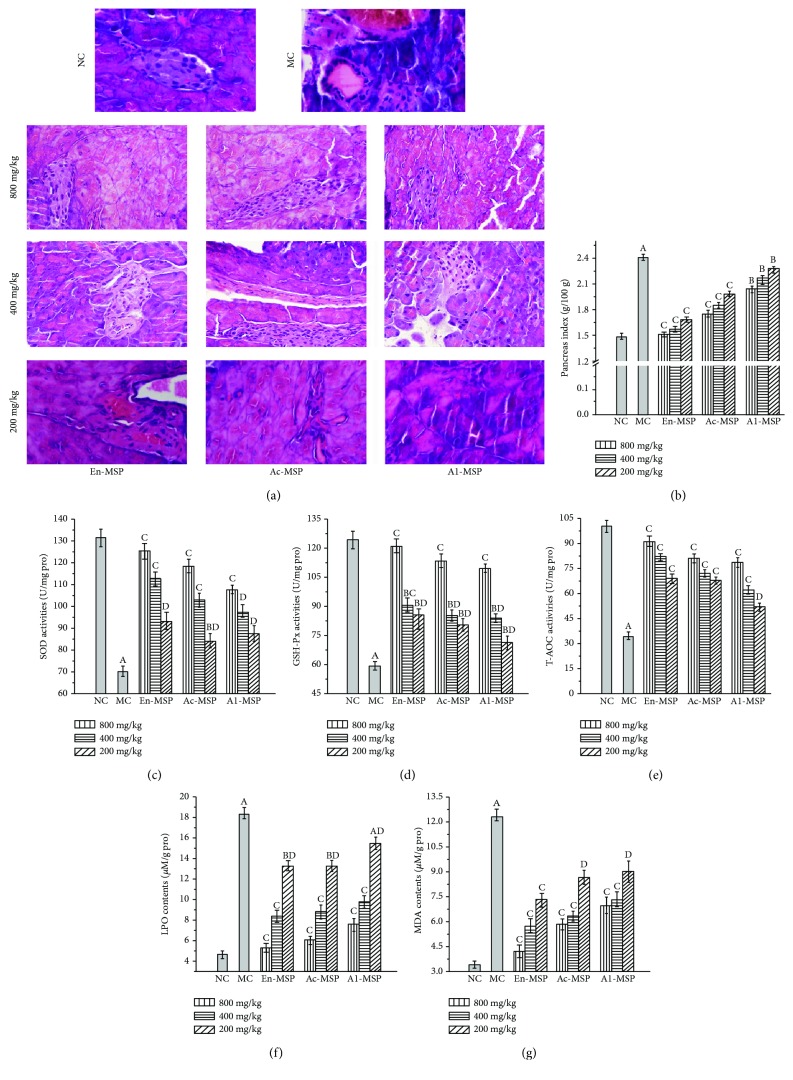
Effects of En-, Ac-, and Al-MSP on the (a) pathological observations, (b) pancreas index, (c–e) pancreatic enzymatic analysis, and (f-g) lipid peroxidation. The values are reported as the means ± SD (*n* = 5). (A): *P* < 0.01 and (B): *P* < 0.05 compared with the NC group, (C): *P* < 0.01 and (D): *P* < 0.05 compared with the MC group.

**Figure 8:**
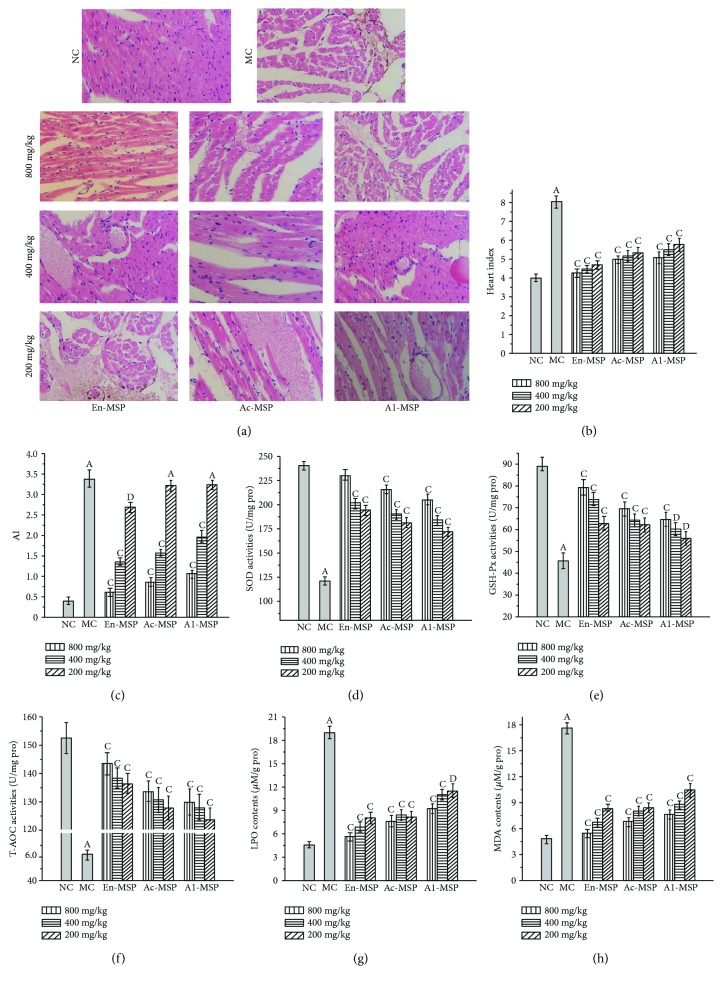
Effects of En-, Ac-, and Al-MSP on the (a) pathological observations, (b) heart index, (c) atherogenic index, (d-f) cardiac enzymatic analysis, and (g-h) lipid peroxidation. The values are reported as the ± SD (*n* = 5). (A): *P* < 0.01 compared with the NC group, (C): *P* < 0.01 and (D): *P* < 0.05 compared with the MC group.

The protective effects of En-, Ac-, and Al-MSP against STZ-induced organic damage (the liver, kidney, pancreas, and heart) were investigated via histopathological observations and measurements of serum parameters, enzymatic activities, and lipid peroxidation contents; the results are shown in Figures 5–8.

Clearly, significant (*P* < 0.05) increases in the liver, kidney, pancreas, and heart indexes were observed in the MC groups compared with the NC groups (Figures 5(b), 6(b), 7(b), and 8(b)), indicating that the organs had suffered serious damage. After treatment with the three polysaccharides, the organic indexes decreased. The effect was especially pronounced for the oral administration of En-MSP at the dosage of 800 mg/kg, after which the indexes of the liver, kidney, pancreas, and heart decreased by 20.28%, 54.36%, 37.34%, and 47.15%, respectively, compared with those in the MC mice, while these indexes decreased by 10.66%, 46.25%, 27.39%, and 38.24% after treatment with Ac-MSP and 11.10%, 37.88%, 15.35%, and 36.88% after administration of Al-MSP in comparison with those in the MC group at the same dose, respectively.

The diabetic mice in the MC group showed severe organic damage in the histopathological observations, as evidenced by architectural changes characteristic of cellular atrophy, nucleus disappearance, loss of membrane integrity, swelling and ballooning degeneration of cells, and inflammatory infiltration. Regarding the unique properties of organs, diabetic mice showed significant liver damage reflecting by lipid accumulation when compared to the NC mice with normal cellular structure (Figure 5(a)). Simultaneously, severe kidney damage, including glomerular degeneration, renal lesions of extracellular matrix deposition, glomerular sclerosis, vacuolation of tubular epithelial cells, and loss of brush border, was observed in STZ-lesioned mice compared with normal mice (Figure 6(a)). For the pancreas, microscopic examinations revealed that STZ injection can cause shrinking and confluent necrosis of pancreatic islets in comparison with the normal rounded appearance of pancreatic islets in the NC mice (Figure 7(a)). Meanwhile, the NC mice showed normal cardiac myocytes and fascicules, while the MC mice showed significant lymphocytic infiltrations, indicating that the heart was dysfunctional (Figure 8(a)). Interestingly, after treatment with En-, Ac-, and Al-MSP at the three tested dosages, the damage was effectively alleviated, as evidenced by improved organ architectures, demonstrating that the polysaccharides had potential effects in preventing DM-induced complications.

Clinically, serum ALT and AST activities are used as biochemical markers for liver damage. As shown in Figures 5(c) and 5(d), significant increases (*P* < 0.05) in serum AST and ALT activities were observed in STZ-induced DM mice compared with the NC mice. Interestingly, after the administration of three polysaccharides, the activities were significantly decreased. The AST and ALT activities were decreased by 58.44% and 68.64% upon treatment with En-MSP at the dosage of 800 mg/kg, while the AST and ALT activities were 47.08% and 60.65% lower after the treatment of Ac-MSP, as well as 40.84% and 46.78% lower after the treatment of Al-MSP compared to the MC group at the same dose. Meanwhile, the results showed that En-, Ac-, and Al-MSP were capable of reversing the increases in serum BUN and CRE levels (clinical markers of kidney damage) in diabetic mice (Figures 6(d) and 6(e)). The levels of BUN and CRE in diabetic mice treated with En-MSP (800 mg/kg) were significantly decreased, by 29.63% and 30.56%, compared with those in the MC mice, while BUN and CRE levels were 30.65% and 29.17% lower in Ac-MSP administration, as well as 34.72% and 18.52% lower in Al-MSP treatment mice than those in the MC mice.

As displayed in Figures 5–8, significant decreases in SOD, GSH-Px, and T-AOC activities, as well as remarkable increases in MDA and LPO contents, were observed in the organs of diabetic mice, indicating that severe oxidative stress had occurred in the liver, kidney, pancreas, and heart. However, the pathological tendency could be ameliorated by oral treatment with polysaccharides. As illustrated in Figures 5(e)–5(g), the hepatic SOD, GSH-Px, and T-AOC activities of mice treated with En-MSP at the dose of 800 mg/kg were increased by 127.50%, 171.09%, and 153.51%, respectively, compared with those of the MC group. Meanwhile, after the administration of En-MSP at the same dose, the activities of SOD, GSH-Px, and T-AOC, respectively, exhibited notable increases of 150.55%, 184.46%, and 171.90% in the kidney, 78.68%, 103.34%, and 165.67% in the pancreas, and 89.91%, 73.47%, and 132.43% in the heart compared with the corresponding levels in the MC group. Additionally, similar tendencies of Ac-MSP and Al-MSP on SOD, GSH-Px, and T-AOC activities were observed in the liver, kidney, pancreas, and heart, respectively. The results indicated that the En-MPS showed stronger antioxidant activities than Ac- and Al-MSP.

Additionally, significant elevations of the MDA and LPO contents were also observed in STZ-induced diabetic mice compared with the NC mice (*P* < 0.05). The results demonstrated that the enhanced oxidative stress in diabetic mice could be counteracted by the three polysaccharides. Briefly, the MDA contents were significantly decreased by 52.12%, 42.00%, 65.53%, and 69.07% in En-MSP treatment mice, by 45.25%, 41.01%, 52.39%, and 61.59% in Ac-MSP administration mice, as well as by 33.33%, 24.66%, 43.39%, and 56.71% in Al-MSP treatment mice than those in the MC mice. For LPO, the contents were remarkably reduced by 42.90%, 66.85%, 70.76%, and 70.22% (En-MSP), by 38.65%, 62.37%, 66.87%, and 59.50% (Ac-MPS), and by 34.08%, 60.33%, 58.71%, and 51.32% (Al-MPS) in the liver, kidney, pancreas, and heart when compared with that in the MC mice at the dose of 800 mg/kg, respectively. Furthermore, the increase in AI values in diabetic mice was significantly inhibited compared with that in the NC mice (Figure 8(c)).

### 3.5. Acute Toxicity Studies

During the entire period of treatment with En-, Ac-, and Al-MSP at a dose of 4000 mg/kg, none of the mice showed any clinical symptoms of toxicity. Furthermore, no death was observed during the procedure or at the end of the period, suggesting that En-, Ac-, and Al-MSP are all essentially nontoxic substances [24].

## 4. Discussion

In the present study, oral administration of three polysaccharides (En-, Ac-, and Al-MSP) significantly decreased GLU levels in STZ-induced diabetic mice, which was also confirmed by Wang et al. [28]. Moreover, the polysaccharides had the potential to improve the weight loss in diabetic mice, and the underlying mechanism may be attributable to the ability of polysaccharides to improve glucose homeostasis [15]. In addition, STZ-induced mice showed serious lipid dysfunction, which was experimentally validated by the increased levels of TG, TC, LDL-C, and VLDL-C, as well as the decreased level of HDL-C, characteristics similar to the clinical properties of human DM. HDL-C plays an important role in carrying cholesterol from peripheral tissues and cells to organs, while LDL-C and VLDL-C can carry cholesterol in the serum to peripheral tissues [29]. This ability was agreed well with the other natural plant product of tomato juice, which had activities of lowing the LDL values and positively attenuating both the glycemia and dyslipidemia against metabolic syndrome in patient [30]. Furthermore, TC and TG are two essential parameters for assessing blood viscosity and the risk of atherosclerosis. Therefore, the results suggested that diabetic mice have rapidly established blood lipid dysfunction and that the oral administration of the three polysaccharides studied has potential effects on improving lipid metabolism.

At present, clinicians and academics have indicated that DM, defined as a systemic metabolic disturbance syndrome, could lead to multiple organ failure [2]. STZ, a highly cytotoxic agent that can induce oxidative stress in organs, has been widely used in establishing DM in animals owing to its favorable properties such as lack of spontaneous remission of hyperglycemia [5, 31]. Although the exact protective mechanism of mushroom polysaccharides against DM and its complications remain poorly understood, accumulated literature has confirmed that its antidiabetes-related activities may involve the antioxidant and preoxidant properties of polysaccharides [32]. Numerous documents have demonstrated that oxidative stress plays an important role in the pathogenesis and progression of diabetes and its complications [1]. A possible mechanism may be that reactive oxygen species (ROS) such as superoxide radicals and hydrogen peroxide could interact with lipids, proteins, and DNA, leading to the dysfunction of these biological macromolecules [33]. In addition, ROS can cause oxidative stress, which can accelerate the damage and destruction of many organs [34, 35]. Previous studies have demonstrated that the accumulation of ROS can induce serious damage to cellular macromolecules, which has proven to be vital in the progress of organ damage and related diseases, including ageing, diabetes mellitus, and hyperglycemia [32]. Hence, investigating the prooxidant-antioxidant status in tissues is quite meaningful for evaluating the antidiabetic effects of polysaccharides. In our present work, a diabetic mouse model under oxidative stress was successfully established by STZ injection and used to assess the protective effects of MSP from *P. eryngii* against DM combined with organic damage (the liver, kidney, pancreas, and heart).

In order to establish the relationship between antioxidant activities and protective effects of the three polysaccharides on STZ-induced liver, heart, pancreas, and kidney damage, the activities of antioxidant enzymes (SOD, GSH-Px, and CAT) and the lipid contents (MDA and LPO) in homogenates of the above tissues were determined. It has been demonstrated that antioxidant enzymes including SOD and GSH-Px have potential protective effects against ROS-mediated tissue damage owing to their ability to decompose superoxide and peroxide and simultaneously block lipid peroxidation [28, 32]. Briefly, SOD catalyses the dismutation of superoxide anions into hydrogen peroxide and oxygen, reducing the intracellular concentrations of superoxide [36]. In addition, as a selenium-containing enzyme, GSH-Px is responsible for the reduction of hydroperoxides and organic peroxides in the presence of reduced glutathione, catalyzing the decomposition of lipid hydroperoxides to their corresponding alcohols and catalyzing the conversion of free hydrogen peroxide to water [37]. Furthermore, lipid peroxidation, a hallmark of oxidative stress, is induced by the interaction between ROS and polyunsaturated fatty acids and leads to the formation of the lipid products MDA and LPO, which are regarded as indexes of cellular damage and cytotoxicity [28]. Extensive evidence has demonstrated that increased lipid peroxidation plays vital roles in the progression of diabetes by altering the transbilayer fluidity gradient and disrupting the normal functions of membrane-bound enzymes and receptors [38]. In this work, a significant decrease in the activities of SOD and GSH-Px and a remarkable increase in MDA and LPO contents were observed in the liver, kidney, pancreas, and heart of STZ-induced diabetic mice, indicating that oxidative stress had occurred. Interestingly, after the administration of the three polysaccharides in our study, a significant increase in enzyme activities and a remarkable decrease in lipid peroxidation were observed in four organs, indicating that polysaccharides had potential protective effects against oxidative stress-derived tissue damage by decreasing lipid peroxidation. Corroborating these results, Wang et al. [28] demonstrated that the polysaccharides from *Inonotus obliquus* can ameliorate the activities of antioxidant enzymes, including CAT, SOD, and GSH-Px activities, in the tissues of diabetic mice. In addition, it has been reported that the medicinal mushroom *Lignosus rhinocerotis* possesses antidiabetic and antioxidant activities, significantly increasing GSH, CAT, and SOD activities and reducing LPO [32].

Clinically, diabetic nephropathy is the most severe complication associated with DM [31]. The kidney is sensitive to exoteric toxic substances and plays vital roles in the glucose metabolism of diabetic animals [31]. Previous reports have indicated that increased serum ALB, CRE, and BUN levels are clinical indicators of kidney status. The suppression of serum ALB, CRE, and BUN levels obtained by treatment with En-, Ac-, and Al-MSP in the present study demonstrated potential nephritic restoration against STZ toxicity.

As another sensitive organ experiencing chemotherapy cytotoxicity *in vivo* and the major site for glucose metabolism in response to insulin, the liver is mainly responsible for maintaining physiological GLU levels by regulating glycolysis and gluconeogenesis [39]. Furthermore, the liver is the main organ site for detoxifying processes as well as clearance of the products of oxidative stress damage [36]. Because ALT localizes to the cytoplasm and AST localizes mainly to organelles, the observed increases in the levels of AST and ALT suggested damage related to increased cell permeability, hepatocyte necrosis, and mitochondrial membranes in diabetic mice [40]. Oral administration of En-, Ac-, and Al-MSP significantly lowered AST and ALT activities, indicating that these polysaccharides had potential effects on improving liver function. These results were in agreement with those for *Catathelasma ventricosum* [41].

Furthermore, low levels of HDL-C and high levels of LDL-C and VLDL-C are the most common lipid abnormalities related to the risk of atherosclerotic cardiovascular diseases and atherosclerotic plaque lesions, which are responses to blood circulation dysfunction in the blood vessel walls [29]. AI is also used as an indicator of protective activity against DM-induced heart diseases. In addition, the decreased GLU level after the oral administration of polysaccharides suggested that organ damage had been alleviated by regulation of GLU levels and improving metabolic disorders.

According to microscopic examinations, the severe hepatic, renal, pancreatic, and cardiac lesions induced by STZ were considerably relieved by administration of En-, Ac-, and Al-MSP, with the morphological appearances in polysaccharide-treated mice being similar to those in the NC mice, suggesting that organ damage could be prevented and repaired by polysaccharide treatment. Similar studies corroborated our conclusion. Das et al. [42] indicated that insulin depletion may result in degenerative structural changes in tissue. Mollazadeh et al. [5] reported that extract of *Euryale ferox* Salisb. improved the histopathology of the pancreas, liver, and kidney in STZ-induced mice.

The antioxidant activities of polysaccharides from edible mushrooms were associated with their molecular characteristics, including monosaccharide abundance compositions, bond types, and molecular weights [43]. Accompanied with present characterizations and animal experiments, the findings were well agreed with the literatures reporting that polysaccharides with small molecular weights had relatively high biological activities [44]. Compared with other reports, Lu et al. [45] reported that *Auricularia auricular* polysaccharides, as heteropolysaccharides, had an effective hypoglycemic effect in diabetic mice. Li et al. [10] indicated that the *P. ferulae* polysaccharides contained 97% glucose and 3% galactose with *α*- and *β*-anomeric structures, which were different from present En-MSP. Ma et al. [13] reported that polysaccharides from *P. eryngii* residues exhibited significant antitumor activity and *β*-type glycosidic linkages were observed by FT-IR. The differences may be related to the stains, culture conditions, and extraction methods. Besides, the En-MSP showed superior antioxidation and attention possibly contributing to the superior physicochemical properties of polysaccharides including good water solubility, high stability, safety, and nontoxicity by enzymatic hydrolysis [46]. Furthermore, Wang et al. [47] had demonstrated that the extractions and modifications could change the three-dimensional structures of polysaccharides, which could be confirmed by SEM and AFM analysis presently. All of the results indicated that En-MSP exhibited potentially superior protective effects against organ damage.

## 5. Conclusions

In the present work, three polysaccharides of En-, Ac-, and Al-MSP were obtained from SMS of *P. eryngii*. Based on the characterizations and *in vivo* animal experiments, the En-MSP with smaller molecular weights, *α*- and *β*-type configurations, integrated surface, and spherical structures showed superior antioxidant and organ protective effects in STZ-induced diabetic mice by increasing the organ enzymatic activities, decreasing the organ lipid peroxide contents, mitigating the serum biochemistry values, and ameliorating the histopathology of diabetic mice. These results could encourage the exploitation and cyclic utilization of SMS from *P. eryngii* and provide new insight into potential mechanisms for the prevention and alleviation of diabetes and its complications.

## Figures and Tables

**Table 1:** Effects of En-, Ac-, and Al-MSP on body weights and GLU levels in STZ-induced diabetic mice (the values are reported as the means ± SD (*n* = 5)).

	GLU (mM)		Body weight (g)	
	Pretreatment	Posttreatment	Pretreatment	Posttreatment
NC	4.22 ± 0.25	4.34 ± 0.18	21.74 ± 2.34	34.83 ± 2.17
MC	14.37 ± 0.74^a^	14.58 ± 0.62^a^	21.36 ± 2.08	23.34 ± 2.11^a^
En-MSP (mg/kg)				
800	14.22 ± 0.55^a^	8.03 ± 0.32^c^	22.34 ± 2.01	28.56 ± 1.76^ac^
400	14.98 ± 0.37^a^	9.41 ± 0.19^c^	21.92 ± 2.15	25.76 ± 2.33^c^
200	14.41 ± 0.21^a^	10.24 ± 0.27^c^	21.78 ± 1.48	24.38 ± 2.21^bc^
Ac-MSP (mg/kg)				
800	14.71 ± 1.03^a^	8.42 ± 0.42^c^	22.01 ± 1.01	27.42 ± 1.58^bc^
400	14.22 ± 0.31^a^	9.26 ± 0.15^c^	22.30 ± 2.21	25.88 ± 2.01^c^
200	14.31 ± 0.34^a^	11.58 ± 0.50^bc^	21.29 ± 2.37	23.08 ± 1.96^ad^
Al-MSP (mg/kg)				
800	14.05 ± 0.91^a^	9.06 ± 0.22^c^	22.76 ± 1.98	26.38 ± 1.89^c^
400	14.99 ± 0.65^a^	10.35 ± 0.61^c^	21.78 ± 2.54	24.16 ± 2.51^bc^
200	14.65 ± 0.34^a^	12.81 ± 0.16^bc^	21.51 ± 1.95	22.99 ± 2.10^c^

^a^ *P* < 0.01 and ^b^*P* < 0.05 compared with the NC group, ^c^*P* < 0.01 and ^d^*P* < 0.05 compared with the MC group.

## Data Availability

The data used to support the findings of this study are available from the corresponding author upon request.

## Abbreviations

Ac-: Acidic-
ALT: Alanine transaminase
ALB: Albumin
Al-: Alkalic-
ANOVA: Analysis of variance
AST: Aspartate aminotransferase
AI: Atherogenic index
AFM: Atomic force microscope
GLU: Blood glucose
CRE: Creatinine
DM: Diabetic mellitus
DMSO: Dimethyl sulfoxide
En-: Enzymatic-
FT-IR: Fourier-transform infrared
GC: Gas chromatography
GSH-Px: Glutathione peroxidase
HDL-C: High-density lipoprotein cholesterol
HPLC: High-performance liquid chromatography
LPO: Lipid peroxide
LDL-C: Low-density lipoprotein cholesterol
MDA: Malondialdehyde
MC: Model control
NC: Normal control
NMR: Nuclear magnetic resonance spectroscopy
Mn: Number average molecular weight
ROS: Reactive oxygen species
SEM: Scanning electron microscope
SMS: Spent mushroom substrate
MSP: Spent mushroom substrate polysaccharides
SD: Standard deviation
STZ: Streptozotocin
SOD: Superoxide dismutases
T-AOC: Total antioxidant capacity
TC: Total cholesterol
TG: Triacylglycerol
UV: Ultraviolet
BUN: Urea nitrogen
VLDL-C: Very low-density lipoprotein cholesterol
Mw: Weight average molecular weight
Mz: Z-average molecular weight.
